# Maternal age-specific risks for adverse birth weights according to gestational weight gain: a prospective cohort in Chinese women older than 30

**DOI:** 10.1186/s12884-023-06231-y

**Published:** 2024-01-05

**Authors:** Yidi Wang, Yunhui Gong, Yujie Xu, Xiaoyu Wang, Shufang Shan, Guo Cheng, Ben Zhang

**Affiliations:** 1 Department of Epidemiology and Biostatistics, Institute of Systems Epidemiology, and West China-PUMC C. C. Chen Institute of Health, West China School of Public Health and West China Fourth Hospital, Sichuan University, Chengdu, China; 2 Department of Gynaecology, West China Women’s and Children’s Hospital, Sichuan University, Chengdu, China; 3 Laboratory of Molecular Translational Medicine, Center for Translational Medicine, Key Laboratory of Birth Defects and Related Diseases of Women and Children (Sichuan University), Department of Pediatrics, West China Women’s and Children’s Hospital, Sichuan University, Chengdu, China

**Keywords:** Gestational weight gain, Older maternal age, Body mass index, Large-for-gestational-age, Small-for-gestational-age

## Abstract

**Background:**

It is unclear whether the effects of abnormal gestational weight gain (GWG) on birth outcomes are differently in women with different maternal ages. This study aimed to investigate maternal age-specific association between GWG and adverse birth weights in Chinese women older than 30.

**Methods:**

19,854 mother-child dyads were selected from a prospective cohort study in Southwest China between 2019 and 2022. Logistic regression model was used to assess the association between GWG, which defined by the 2009 Institute of Medicine guidelines, and adverse birth weights including large- and small-for-gestational-age (LGA and SGA), stratified by maternal age (31–34 years and ≥ 35 years).

**Results:**

In both maternal age groups, excessive and insufficient GWG were associated with increased odds of LGA and SGA, respectively. After women were categorized by pre-pregnancy body mass index, the associations remained significant in women aged 31–34 years, whereas for women aged ≥ 35 years, the association between excessive GWG and the risk of LGA was only significant in normal weight and overweight/obese women, and the significant effect of insufficient GWG on the risk of SGA was only observed in underweight and overweight/obese women. Moreover, among overweight/obese women, the magnitude of the association between insufficient GWG and the risk of SGA was greater in those aged ≥ 35 years (31–34 years: OR 2.08, 95% CI 1.19–3.55; ≥35 years: OR 2.65, 95% CI 1.47–4.74), while the impact of excessive GWG on the risk of LGA was more pronounced in those aged 31–34 years (31–34 years: OR 2.18, 95% CI 1.68–2.88; ≥35 years: OR 1.71, 95% CI 1.30–2.25).

**Conclusions:**

The stronger associations between abnormal GWG and adverse birth weights were mainly observed in women aged 31–34 years, and more attention should be paid to this age group.

**Supplementary Information:**

The online version contains supplementary material available at 10.1186/s12884-023-06231-y.

## Background

Excessive or insufficient gestational weight gain (GWG) has been associated with a wide range of adverse perinatal outcomes, including large-for-gestational-age (LGA) [1] and small-for-gestational-age (SGA) [2]. Many studies have reported that LGA and SGA are associated with early morbidity and mortality [3, 4] as well as an increased risk of chronic health conditions such as obesity [5], diabetes [5], and hypertension [6] later in life. Therefore, maintaining an appropriate weight during pregnancy is important. Many factors may affect GWG, among which maternal age receives much attention as a trend in delayed childbearing has been observed in many countries [7, 8]. Advanced maternal age (AMA) is commonly defined as pregnancy at the age of 35 and older [9]. In China, the fertility rate for women aged 35–39 years was 5.7‰ in 1995 and rose to 18.6‰ in 2015 [10]. Furthermore, the number of women aged ≥ 35 years has increased from 8.5 to 13.5% after the announcement of the two-child policy [11]. Older women usually gain less weight during pregnancy [12], and they are at a higher risk for a range of adverse perinatal outcomes, given that women aged 35–39 years had a 1.31-fold increased risk for LGA compared to those aged 25–29 years [13], and the SGA risk was increased 1.46-fold in women older than 40 years compared with women aged < 35 years [14]. Considering these age-related differences, it is of interest to know whether the association between GWG and adverse birth weight still exists in women of AMA, however, such evidence is scarce.

Although the age ≥ 35 years has been suggested to be the cut-off for increased risk of adverse perinatal outcomes, some studies have reported that the risks were evident in women as early as 30–34 years by comparing with those aged 20–29 years [13, 15]. The relationship between GWG and adverse birth weights has been examined in women with mean maternal age at 30–34 years [1, 16–20], but these studies were limited by either small sample size [1, 16, 18–20] or retrospective design [16, 17, 19], and most of them were conducted in Western countries [1, 17–20]; little is known about the impact of GWG in Chinese women older than 30. Over the last two decades, the fertility rate for Chinese women aged 30–34 has risen from 26.5‰ to 45.3‰ (1995–2015), whereas it declined for women aged below 30 [10]. The mean age at childbearing in China has increased by 3.3 years during the same period (25.2 years in 1995 to 28.5 years in 2015) [10]. With the relaxation of the child policy, the mean childbearing age has increased to 29.6 years in 2019–2020 [21] and may exceed 30 years in the near future. Considering the increasing trend in women who give birth over 30, a prospective cohort with a large sample size to explore the role of GWG on adverse birth weights in this older Chinese population but not yet considered as AMA and then compare with those of AMA may have important implications.

Using the large prospective cohort from Southwest China, this study thus aimed to investigate the age-specific association between GWG and adverse birth weights in women above 30.

## Methods

### Study population

This prospective cohort study was conducted in the three provinces of Southwest China (Sichuan, Yunnan, and Guizhou provinces) between 2019 and 2022, aiming to investigate the effect of maternal nutritional status concerning pre-pregnancy body mass index (BMI) and GWG, on maternal and neonatal health outcomes in older mothers. Using the method of multistage sampling, 18 public hospitals and community health care centers were randomly selected from 12 urban and rural areas (1–2 hospitals in each area) in the three provinces. Pregnant women at their first prenatal visit (9^+ 0^-11^+ 6^ gestational week) were invited to participate if they were older than 30 years of age, singleton pregnancy, and had lived in their current residence for at least one year. Women with preexisting conditions such as diabetes, hypertension or other major diseases were excluded. Each pregnant women was scheduled to visit obstetricians (every 4 weeks through 25 weeks of gestation, every 2 weeks from 26 to 33 weeks of gestation, and weekly thereafter until birth) for medical examination and anthropometric measurements. Study participants were followed up at each prenatal visit until they gave birth. The study was approved by the Ethics Committee of the Sichuan University. Written informed consent was obtained from all participants.

During 2019 to 2022, there were 21,521 women recruited in this study. Pregnant women without information on pre-pregnancy or end pregnancy weight, neonatal outcomes or having stillbirth were excluded, resulting in a total of 19,854 mother-child dyads in the analysis of total GWG. Additional measure of GWG rate in the second and third trimesters (*n* 12,801) was used to assess the robustness of the results. The flow chart of the participants is shown in Figure S1.

### Maternal measurements

Basic information including socio-demographic characteristics, medical history, family history of chronic diseases, and pre-pregnancy body weight was collected through a self-administrated questionnaire as well as women’s height was measured during the first prenatal visit. Pre-pregnancy BMI was calculated [weight (kg) / height (m^2^)], and was further categorized into three groups according to World Health Organization Asian BMI classification [22]: underweight (< 18.5 kg/m^2^), normal weight (18.5–22.9 kg/m^2^), and overweight/obese (≥ 23 kg/m^2^).

Anthropometric measures including maternal body weight during pregnancy was measured at each prenatal visit, and information on pregnancy complications was obtained from the Medical Birth Registry. GWG was calculated as the difference between the latest weight before delivery and pre-pregnancy weight. Women were categorized as less than, within, or greater than the 2009 Institute of Medicine (IOM)’s pre-pregnancy BMI-specific GWG recommendation [23].

To assess the robustness of the results, the rate of GWG in the second and third trimesters was estimated as [the difference between the first weight recorded in the second trimester (12^+ 1^-15^+ 6^ gestational week) and the last weight recorded before delivery] / (gestational age − 13)] [23]. The rate of GWG in the second and third trimesters was classified following the 2009 IOM recommendation [23].

### Birth weights

Birth weight was measured by trained medical workers and were retrieved from the Medical Birth Registry. LGA and SGA were defined as birth weight above the 90th percentile or below the 10th percentile, respectively, after adjusting for gender and gestational age according to the Chinese neonatal birth weight curve [24]. Gestational age was estimated based on the women’s last menstrual period and was confirmed with ultrasound examination.

### Statistical analysis

Continuous variables were presented as median with 25% and 75% quartiles as they were not normally distributed, and categorical variables were reported as frequency and proportion. The Chi-square test and Wilcoxon test were used to compare maternal and neonatal characteristics between subgroups. Logistic regression was conducted to estimate the unadjusted odd ratios (OR), adjusted OR, and 95% confidence intervals (CI) of adverse birth weights across GWG categories. GWG within the IOM recommendation was used as a reference group. Variables that were risk factors for adverse birth weights based on literature [14, 25–27] and were statistically different according to maternal GWG (Table S1) were identified as potential confounders: maternal age, pre-pregnancy BMI, gestational diabetes mellitus (GDM), preeclampsia, caesarean delivery, gestational age, and neonatal gender. Each potential confounder was added in the model one at a time, and the confounder was kept in the model if the changes of estimates were greater than 10% [28]. As a result, the association of GWG with LGA and SGA was adjusted for maternal age, pregnancy BMI, GDM, and preeclampsia. As there was an interaction effect between GWG and pre-pregnancy BMI on adverse birth weights (*P* < 0.05), subgroup analyses stratified by pre-pregnancy BMI category were performed in the same manner as above described.

Since the traditional cut-off age for AMA was 35 years [9], participants were further categorized into two maternal age groups: 31–34 years (*n* 12,189) and ≥ 35 years (*n* 7665), in order to examine the age-specific association between GWG and adverse birth weights. Logistic regression stratifying by maternal age was performed, and models for LGA and SGA were adjusted for pregnancy BMI, GDM, and preeclampsia.

To test the robustness of the results, the same analysis was repeated by using GWG rate in the second and third trimesters, and the adequacy of GWG rates was defined based on the 2009 IOM recommendation [23]. All analyses were performed using SAS 9.3. *P* < 0.05 was considered as statistically significant.

A post-hoc power test (SAS proc power procedure) showed that the power was > 0.999 for the impact of abnormal GWG on adverse birth weight (*n* 19,854), and was > 0.999 when the analysis was stratified by maternal age group (*n* 12,189 for 31–34 years and *n* 7665 for ≥ 35 years). The power was higher than the criteria (0.8) suggested by Cohen [29], indicating that the number of participants enrolled was sufficient.

## Results

### General characteristics of the study population

The general characteristics of the total participants and characteristics according to maternal age are listed in Table 1. A total of 19,854 women were included in this study with an average age of 34.0 years and gained 12.5 kg over pregnancy. 9.9% of women were classified as underweight and 27.5% were overweight/obese before pregnancy. After stratified by maternal age, women aged 35 years and older were more likely to be overweight/obese before pregnancy, but gained less weight during pregnancy than those in the 31–34 years age group (all *P* < 0.0001). Compared with the 31–34 years age group, the prevalence of GDM, preeclampsia, caesarean delivery, and LGA were significantly higher in the ≥ 35 years age group (all *P* < 0.05). There were no significant differences in the prevalence of SGA between two maternal age groups.

**Table 1:** General characteristics of the study participants according to maternal age

Characteristics	Total (n = 19,854)	Maternal age		*P-value*
		31–34 yr (n = 12,189)	≥ 35 yr (n = 7665)	
**Mothers**				
Pre-pregnancy BMI (kg/cm^2^)^a^	21.3 (19.7, 23.2)	21.1 (19.5, 22.9)	21.7 (20.1, 23.5)	< 0.0001
Underweight (n (%))	1965 (9.9)	1422 (11.7)	543 (7.1)	< 0.0001
Normal weight (n (%))	12,477 (62.8)	7812 (64.1)	4665 (60.9)	
Overweight/obese (n (%))	5412 (27.3)	2955 (24.2)	2457 (32.1)	
Gestational weight gain (kg)	12.5 (10.0, 15.0)	13.0 (10.0, 15.0)	12.0 (9.5, 15.0)	< 0.0001
Rate of second and third trimester weight gain (kg/wk)	0.46 (0.36, 0.55)	0.47 (0.38, 0.56)	0.44 (0.34, 0.54)	< 0.0001
Gestational diabetes mellitus (n (%))	5193 (26.2)	2800 (23.0)	2393 (31.2)	< 0.0001
Preeclampsia (n (%))	349 (1.8)	180 (1.5)	169 (2.2)	< 0.0001
Caesarean delivery (n (%))	13,723 (69.1)	7754 (63.6)	5969 (77.9)	< 0.0001
**Newborns**				
Gestational age at delivery (weeks)	39.1 (38.7, 39.7)	39.3 (38.7, 39.9)	39.0 (38.5, 39.4)	< 0.0001
Gender, females (n (%))	9640 (48.6)	5889 (48.3)	3751 (48.9)	0.39
Birth length (cm)	50.0 (49.0, 51.0)	50.0 (49.0, 51.0)	50.0 (48.0, 51.0)	0.0002
Birth weight (g)	3300 (3035, 3570)	3300 (3040, 3570)	3300 (3030, 3570)	0.38
Preterm birth (n (%))	881 (4.4)	490 (4.0)	391 (5.1)	0.0003
Macrosomia (n (%))	926 (4.7)	575 (4.7)	351 (4.6)	0.65
Large for gestational age (n (%))	1592 (8.0)	936 (7.7)	656 (8.6)	0.03
Small for gestational age (n (%))	1133 (5.7)	725 (6.0)	408 (5.3)	0.06

Data are expressed as median (25%, 75%) or n (%)

^a^ Pre-pregnancy BMI was based on WHO Asians [22]

Maternal and neonatal characteristics according to GWG category are presented in Table S1. 27.4% of the women gained weight below the IOM recommendation and 31.2% gained weight above the recommendation. Women with excessive weight gain were more likely to develop GDM and preeclampsia, and deliver LGA infants, whereas women with inadequate weight gain were more likely to deliver SGA infants (all *P* < 0.0001).

### The association between GWG and birth weights

The association of GWG with LGA and SGA for the whole study population is shown in Table 2. Compared to women who gained weight within the IOM recommendation, those gained excessive weight had higher risks of delivering LGA infants (OR 1.77, 95% CI 1.57–1.99, *P* < 0.0001), and lower risk of delivering SGA infants (OR 0.72, 95% CI 0.60–0.85, *P* < 0.05). Conversely, women who gained insufficient weight had higher risks of delivering SGA infants (OR 1.63, 95% CI 1.42–1.86, *P* < 0.0001), and lower risk of delivering LGA infants (OR 0.48, 95% CI 0.40–0.57, *P* < 0.0001). The associations for LGA remained significant after the women were categorized into different pre-pregnancy BMI groups, while the protective effect of excessive GWG on SGA was only observed in normal weight women.

**Table 2:** Association between gestational weight gain and adverse birth weights according to the IOM guidelines in total cohort

GWG	LGA			SGA		
	n (%)	Crude OR (95% CI)	Adjusted OR (95% CI)	n (%)	Crude OR (95% CI)	Adjusted OR (95% CI)
**Overall** ^a^ **(n = 19,854)**						
Below	183 (3.4)	0.47 (0.39, 0.55)	0.48 (0.40, 0.57) ^**^	472 (8.7)	1.67 (1.46, 1.91)	1.63 (1.42, 1.86) ^**^
Within	571 (7.0)	1.00	1.00	441 (5.4)	1.00	1.00
Above	838 (13.5)	2.09 (1.87, 2.34)	1.77 (1.57, 1.99) ^**^	220 (3.6)	0.65 (0.55, 0.76)	0.72 (0.60, 0.85) ^*^
**Underweight** ^**b**^ **(n = 1965)**						
Below	5 (0.7)	0.20 (0.07, 0.46)	0.19 (0.07, 0.46) ^*^	110 (15.3)	2.08 (1.53, 2.83)	2.02 (1.48, 2.76) ^**^
Within	34 (3.4)	1.00	1.00	79 (8.0)	1.00	1.00
Above	19 (7.5)	2.26 (1.25, 4.00)	2.32 (1.28, 4.11) ^*^	19 (7.5)	0.93 (0.54, 1.53)	0.92 (0.53, 1.51)
**Normal weight** ^**b**^ **(n = 12,477)**						
Below	152 (3.6)	0.51 (0.42, 0.62)	0.49 (0.41, 0.60) ^**^	316 (7.6)	1.44 (1.22, 1.70)	1.44 (1.22, 1.71) ^**^
Within	376 (6.9)	1.00	1.00	293 (5.4)	1.00	1.00
Above	332 (11.7)	1.78 (1.52, 2.08)	1.81 (1.55, 2.11) ^**^	98 (3.4)	0.63 (0.49, 0.79)	0.61 (0.48, 0.76) ^**^
**Overweight/Obese** ^**b**^ **(n = 5412)**						
Below	26 (4.7)	0.50 (0.32, 0.74)	0.47 (0.30, 0.71) ^*^	46 (8.4)	2.24 (1.52, 3.29)	2.32 (1.55, 3.44) ^**^
Within	161 (9.1)	1.00	1.00	69 (3.9)	1.00	1.00
Above	487 (15.8)	1.87 (1.55, 2.26)	1.94 (1.60, 2.36) ^**^	103 (3.3)	0.85 (0.62, 1.16)	0.76 (0.56, 1.05)

^a^ Adjusted for maternal age, pre-pregnancy BMI, gestational diabetes mellitus, and preeclampsia

^b^ Adjusted for maternal age, gestational diabetes mellitus, and preeclampsia

^*^P < 0.05, ^**^P < 0.0001

Abbreviations: IOM, Institute of Medicine; GWG, gestational weight gain; LGA, large-for-gestational-age; SGA, small-for-gestational-age

### Maternal age-specific association between GWG and birth weights

**Fig. 1:**
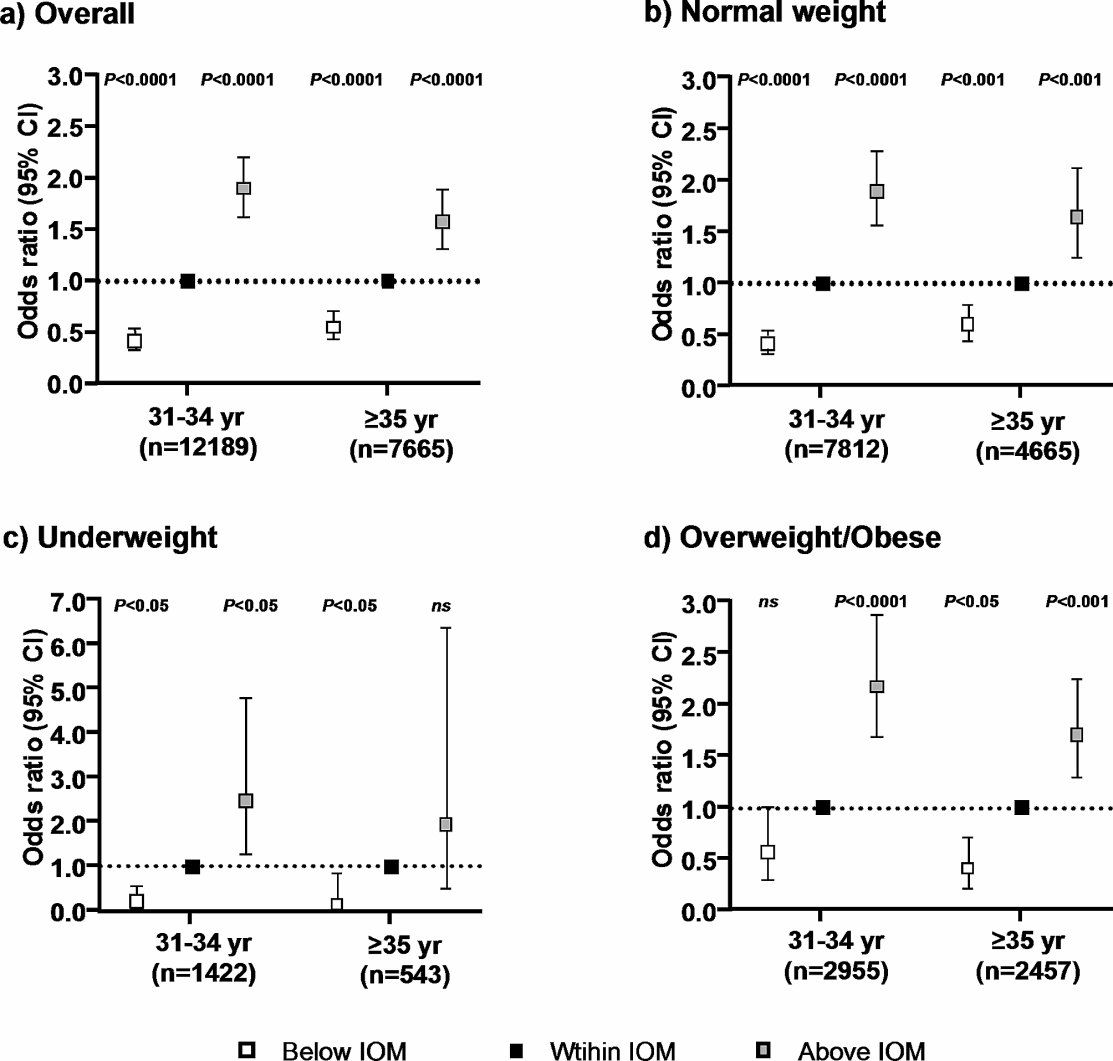
Age-specific association between gestational weight gain and large-for-gestational-age according to the IOM guidelines **a**) Models were adjusted for pre-pregnancy BMI, gestational diabetes mellitus, and preeclampsia; **b**-**d**) Models were adjusted for gestational diabetes mellitus and preeclampsia

**Fig. 2:**
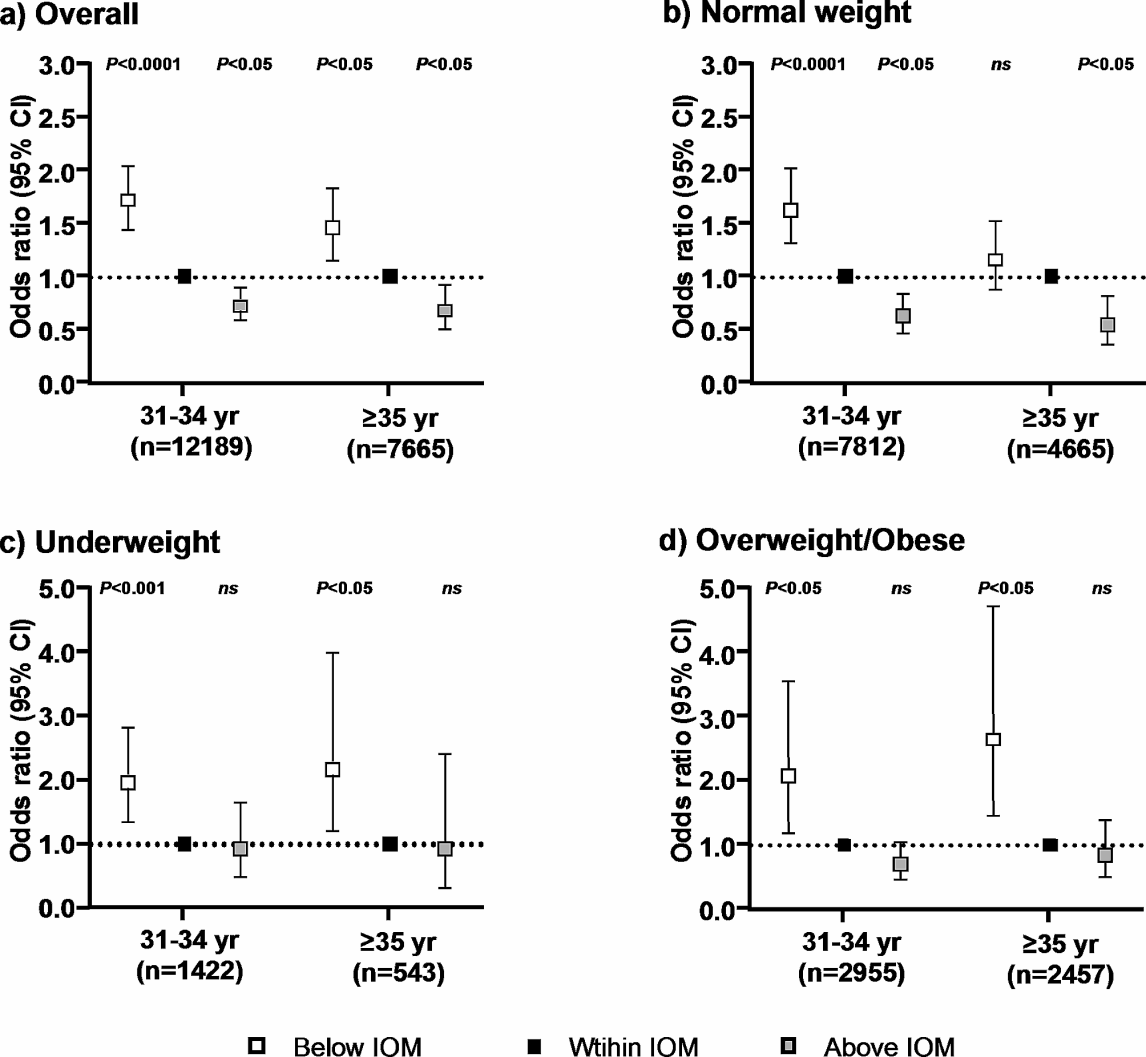
Age-specific association between gestational weight gain and small-for-gestational-age according to the IOM guidelines **a**) Models were adjusted for pre-pregnancy BMI, gestational diabetes mellitus, and preeclampsia; **b**-**d**) Models were adjusted for gestational diabetes mellitus and preeclampsia

The assessment of GWG on the risks of LGA and SGA was further performed in two maternal age groups (Figs. 1 and 2). In both maternal age groups, excessive weight gain was associated with an increased risk of LGA (31–34 years: OR 1.90, 95% CI 1.63–2.21, *P* < 0.0001; ≥35 years: OR 1.58, 95% CI 1.31–1.90, *P* < 0.0001). After the women were categorized into different pre-pregnancy BMI groups, the association remained significant in the 31–34 years age group, whereas for ≥ 35 years age group, the association between excessive GWG and the risk of LGA was only significant in normal weight and overweight/obese women. Moreover, among the overweight/obese women, the effect of excessive GWG on the risk of LGA was more pronounced in the younger age group (31–34 years: OR 2.18, 95% CI 1.68–2.88, *P* < 0.0001; ≥35 years: OR 1.71, 95% CI 1.30–2.25, *P* < 0.001).

Women who gained weight below the IOM recommendation was associated with a higher risk of SGA in both maternal age groups (31–34 years: OR 1.72, 95% CI 1.45–2.04, *P* < 0.0001; ≥35 years: OR 1.46, 95% CI 1.16–1.83, *P* < 0.05). After stratified by pre-pregnancy BMI, while inadequate GWG was still a risk factor for SGA in all BMI categories in the 31–34 years age group, for ≥ 35 years age group, the significant association was only observed in women who were underweight and overweight/obese. Moreover, among the overweight/obese women, the magnitude of the association between inadequate GWG and higher risk of SGA was greater for older age group (31–34 years: OR 2.08, 95% CI 1.19–3.55, *P* < 0.05; ≥35 years: OR 2.65, 95% CI 1.47–4.74, *P* < 0.05).

### Association between GWG rate in the second and third trimesters and birth weights

The rate of GWG in the second and third trimesters was further estimated. The proportions of women with inadequate, adequate, and excessive GWG rate were 17.6%, 33.5%, and 48.9%, respectively. Results for GWG rate were generally consistent with total GWG regarding LGA and SGA (Table S2 and Table S3), suggesting that stronger associations between abnormal GWG and adverse birth weights were mainly observed in women aged 31–34 years.

## Discussion

The present study demonstrated that among overweight and obese women, the effect of insufficient GWG on the risk of SGA was greater with increasing maternal age, whereas the association between excessive GWG and the risk of LGA was more evident in those aged 31–34 years. Moreover, the effect of abnormal GWG on the risk of LGA and SGA was not observed in underweight and normal weight women aged ≥ 35 years, respectively.

Prospective cohort studies of GWG and adverse birth weights in older Chinese women have been scarce. The present study was conducted in this older population (31–34 years and ≥ 35 years), confirming the association between excessive GWG and higher risk of LGA, but also showing that the effect was more pronounced in women aged 31–34 years, especially for those who were underweight and overweight/obese before pregnancy. These results are likely in part attributable to the variations in the amount of weight gained during pregnancy between two maternal age groups, given that women who aged 31–34 years had more GWG than those aged ≥ 35 years. A higher proportion of overweight and obese women in the ≥ 35 years age group than in the 31–34 years age group may partially account for the difference in GWG between two groups. Overweight and obese women usually receive greater attention from gynecologists and obstetricians due to their higher risks of adverse perinatal outcomes [17, 30, 31] and are recommended to gain less weight during pregnancy [23].

The present study did not include women aged ≤ 30 years, however, it is noticed that the risk estimate of excessive GWG on LGA in women aged 31–34 years of the present study was higher than most of studies that conducted in Chinese women with mean maternal age at 25–30 years (OR 1.90 vs. OR 1.42–1.70) [32–34], even though their GWG was greater. Considering that some studies found that the risks of LGA were similar in women aged 31–34 years and ≥ 35 years [13, 15], it is speculated that maternal age had a higher effect than GWG on the risk of LGA when comparisons were made between women aged 25–30 and 31–34 years, whereas GWG had more influence on LGA when comparisons were made between 31 and 34 years and ≥ 35 years. While further research with a wide maternal age category is warranted to verify these results and to explore the underlying mechanisms, the present study emphasizes the need to increase attention to women with childbearing age at 31–34 years, who are becoming increasingly prevalent in China but have been neglected since the commonly used definition of AMA is ≥ 35 years.

Unlike LGA, the effect of insufficient GWG on the risk of SGA was more evident for those aged ≥ 35 years among overweight and obese women in this study. Given that the risk estimate of insufficient GWG on SGA in prior research that conducted in overweight/obese Chinese women with mean maternal age < 30 years was smaller than that of the present study (OR 1.18–1.40 vs. OR 2.32) [31, 35], it seems like that the effect of insufficient GWG on the risk of SGA in overweight/obese women was greater with increasing maternal age. This observation, however, was not seen in other pre-pregnancy BMI categories. Overweight and obesity is a complex metabolic state, and evidence has indicated that overweight/obesity is not only associated with fetal overgrowth but also increases the risk of SGA [2] despite of others have reported inconsistent results [30, 36]. The impaired placental function and abnormal transfer of nutrients through placenta in overweight and obese women was regarded as one of the possible mechanisms [37]. Considering that older maternal age is also closely related with placental defects [38], it is likely that some connections may exist between older maternal age and maternal overweight/obesity which exacerbate the effect of insufficient GWG on SGA, but further investigation is required to confirm such a possibility.

It is worth noting that the adverse effect of insufficient GWG on the risk of SGA was not observed in normal weight women aged ≥ 35 years. Although the reasons behind it are uncertain, this finding suggests that both maternal age and pre-pregnancy BMI influence the association between GWG and adverse birth weights. The current data again point out the necessity to focus more on women aged 31–34 years, given that the stronger associations between abnormal GWG and adverse birth weights were mainly observed in this age group.

Overall, the present study showed that older women with excessive or insufficient GWG do not appear to be at the same risk for adverse birth weights compared to their younger counterparts. Recommending a single optimal GWG range for women of different maternal ages might be inappropriate. To date, very limited study [39] has been conducted to investigate the optimal weight gain for women with different maternal ages. The current findings may stimulate future investigation to improve the current GWG guidelines by considering different characteristics (e.g. maternal age) of women. In addition, this study highlights the need for healthcare professionals to increase concerns about excessive GWG and LGA in women aged 31–34 years, given that this population is increasing in China and infants born with LGA are closely associated with childhood obesity that has become a serious health problem in the world.

This was the first study to investigate the associations between GWG and adverse birth weights stratified by pre-pregnancy BMI in older Chinese women, and the results were then compared in women aged 31–34 years and those of AMA. The findings may contribute to the limited information available on this population by highlighting the importance of GWG as well as pre-pregnancy BMI on neonatal growth and development. The large sample size, prospective design, and rigorous collection of data allowed a valid assessment of GWG on the risks of adverse birth weights. Moreover, additional measure of GWG rate in the second and third trimesters was performed to control the effect of the length of gestation on adverse birth weights.

Limitations of the present study should be noted. Pre-pregnancy weight was self-reported which might result in recall error, however, some evidence has indicated that self-reported and clinically measured pre-pregnancy weights were highly correlated [40]. While the number of overall study participants was large, the sample size in the subpopulation such as underweight women, overweight or obese women aged ≥ 35 years was limited. Because of this reason, the overweight and obese women were analyzed together. Further research with a large sample size in different maternal ages and pre-pregnancy BMIs is required to assess the association between GWG and adverse birth weights.

## Conclusions

In conclusion, the present study demonstrated that maternal age might influence the association between GWG and adverse birth weights in Chinese older women. More attention should be paid to those aged 31–34 years, who have been neglected but their risks of adverse perinatal outcomes were as high as in those of AMA. More importantly, the stronger associations between abnormal GWG and adverse birth weights were mainly seen in the age group of 31–34 years. Future research on association of GWG with other perinatal outcomes such as GDM, preeclampsia and preterm birth in women of different maternal ages are needed to confirm this conclusion, and the results will help to determine whether women of different maternal ages warrant separate GWG recommendations.

### Electronic supplementary material

Below is the link to the electronic supplementary material.

Supplementary Material 1

## Data Availability

The datasets used and/or analyzed during the current study are available from the corresponding author on reasonable request.

## Abbreviations

AMA: Advanced maternal age
BMI: Body mass index
CI: Confidence intervals
GDM: Gestational diabetes mellitus
GWG: Gestational weight gain
IOM: Institute of Medicine
LGA: Large-for-gestational-age
OR: Odds ratio
SGA: Small-for-gestational-age

## References

[1] Badon SE, Dyer AR, Josefson JL, HAPO Study Cooperative Research Group (2014). Gestational weight gain and neonatal adiposity in the hyperglycemia and adverse pregnancy outcome study-north American region. Obes (Silver Spring).

[2] Liu L, Hong Z, Zhang L (2015). Associations of prepregnancy body mass index and gestational weight gain with pregnancy outcomes in nulliparous women delivering single live babies. Sci Rep.

[3] Pasupathy D, McCowan LME, Poston L, Kenny LC, Dekker GA, North RA (2012). Perinatal outcomes in large infants using customised birthweight centiles and conventional measures of high birthweight. Paediatr Perinat Epidemiol.

[4] Horbar JD, Carpenter JH, Badger GJ, Kenny MJ, Soll RF, Morrow KA (2012). Mortality and neonatal morbidity among infants 501 to 1500 grams from 2000 to 2009. Pediatrics.

[5] Johnsson IW, Haglund B, Ahlsson F, Gustafsson J (2015). A high birth weight is associated with increased risk of type 2 Diabetes and obesity. Pediatr Obes.

[6] Prinz N, Putri RR, Reinehr T, Danielsson P, Weghuber D, Norman M (2023). The association between perinatal factors and cardiometabolic risk factors in children and adolescents with overweight or obesity: a retrospective two-cohort study. PLoS Med.

[7] Sheen JJ, Wright JD, Goffman D, Kern-Goldberger AR, Booker W, Siddiq Z (2018). Maternal age and risk for adverse outcomes. Am J Obstet Gynecol.

[8] Londero AP, Rossetti E, Pittini C, Cagnacci A, Driul L (2019). Maternal age and the risk of adverse pregnancy outcomes: a retrospective cohort study. BMC Pregnancy Childbirth.

[9] Laopaiboon M, Lumbiganon P, Intarut N, Mori R, Ganchimeg T, Vogel JP (2014). Advanced maternal age and pregnancy outcomes: a multicountry assessment. BJOG.

[10] Yang S, Jiang Q, Sanchez-Barricarte JJ (2022). China’s fertility change: an analysis with multiple measures. Popul Health Metr.

[11] Li HT, Xue M, Hellerstein S, Cai Y, Gao Y, Zhang Y (2019). Association of China’s universal two child policy with changes in births and birth related health factors: national, descriptive comparative study. BMJ.

[12] Hawley NL, Johnson W, Hart CN, Triche EW, Ah Ching J, Muasau-Howard B (2015). Gestational weight gain among American Samoan women and its impact on delivery and infant outcomes. BMC Pregnancy Childbirth.

[13] Cao J, Xu W, Liu Y, Zhang B, Zhang Y, Yu T (2022). Trends in maternal age and the relationship between advanced age and adverse pregnancy outcomes: a population-based register study in Wuhan, China, 2010–2017. Public Health.

[14] Khalil A, Syngelaki A, Maiz N, Zinevich Y, Nicolaides KH (2013). Maternal age and adverse pregnancy outcome: a cohort study. Ultrasound Obstet Gynecol.

[15] Kenny LC, Lavender T, McNamee R, O’Neill SM, Mills T, Khashan AS (2013). Advanced maternal age and adverse pregnancy outcome: evidence from a large contemporary cohort. PLoS ONE.

[16] Zheng W, Huang W, Zhang Z, Zhang L, Tian Z, Li G (2019). Patterns of gestational weight gain in women with overweight or obesity and risk of large for gestational age. Obes Facts.

[17] Enomoto K, Aoki S, Toma R, Fujiwara K, Sakamaki K, Hirahara F (2016). Pregnancy outcomes based on pre-pregnancy body mass index in Japanese women. PLoS ONE.

[18] Lan-Pidhainy X, Nohr EA, Rasmussen KM (2013). Comparison of gestational weight gain-related pregnancy outcomes in American primiparous and multiparous women. Am J Clin Nutr.

[19] Bianchi C, de Gennaro G, Romano M, Aragona M, Battini L, Del Prato S (2018). Pre-pregnancy obesity, gestational Diabetes or gestational weight gain: which is the strongest predictor of pregnancy outcomes?. Diabetes Res Clin Pract.

[20] Sámano R, Chico-Barba G, Flores-Quijano ME, Godinez-Martinez E, Martiinez-Rojano H, Ortiz-Hernandez (2021). Association of pregestational BMI and gestational weight gain with maternal and neonatal outcomes in adolescents and adults from Mexico City. Int J Environ Res Public Health.

[21] National Bureau of Statistics of China. China Population Census Yearbook 2020. China Statistics Press; 2022.

[22] WHO Expert Consultation (2004). Appropriate body-mass index for Asian populations and its implications for policy and intervention strategies. Lancet.

[23] Institute of Medicine (US) and National Research Council (US) Committee to Reexamine IOM Pregnancy Weight Guidelines. In: Rasmussen KM, Yaktine AL, editors. Weight gain during pregnancy: reexamining the guidelines. National Academies Press; 2009. 20669500

[24] Zhu L, Zhang R, Zhang S, Shi W, Yan W, Wang X (2015). [Chinese neonatal birth weight curve for different gestational age]. Zhonghua Er Ke Za Zhi = Chinese Journal of Pediatrics.

[25] Sun Y, Shen Z, Zhan Y, Wang Y, Ma S, Zhang S (2020). Effects of pre-pregnancy body mass index and gestational weight gain on maternal and infant Complications. BMC Pregnancy Childbirth.

[26] Ye W, Luo C, Huang J, Li C, Liu Z, Liu F (2022). Gestational Diabetes Mellitus and adverse pregnancy outcomes: systematic review and meta-analysis. BMJ.

[27] Harvey L, van Elburg R, van der Beek EM (2021). Macrosomia and large for gestational age in Asia: one size does not fit all. J Obstet Gynaecol Res.

[28] Maldonado G, Greenland S (1993). Simulation study of confounder-selection strategies. Am J Epidemiol.

[29] Cohen J (1992). A power primer. Psychol Bull.

[30] Du MK, Ge LY, Zhou ML, Ying J, Qu F, Dong MY (2017). Effects of pre-pregnancy body mass index and gestational weight gain on neonatal birth weight. J Zhejiang Univ Sci B.

[31] Liu X, Wang H, Yang L, Zhao M, Magnussen CG, Xi B (2022). Associations between gestational weight gain and adverse birth outcomes: a population-based retrospective cohort study of 9 million mother-infant pairs. Front Nutr.

[32] Wang Y, Ma H, Feng Y, Zhan Y, Wu S, Cai S (2020). Association among pre-pregnancy body mass index, gestational weight gain and neonatal birth weight: a prospective cohort study in China. BMC Pregnancy Childbirth.

[33] Zhao R, Xu L, Wu ML, Huang SH, Cao XJ (2018). Maternal pre-pregnancy body mass index, gestational weight gain influence birth weight. Women Birth.

[34] Wang T, Li L, Wu C (2021). Body mass index and gestational weight gain are associated with maternal and neonatal outcomes based on Chinese women. J Diabetes Res.

[35] Li C, Liu Y, Zhang W (2015). Joint and Independent associations of gestational weight gain and pre-pregnancy body mass index with outcomes of pregnancy in Chinese women: a retrospective cohort study. PLoS ONE.

[36] Dzakpasu S, Fahey J, Kirby RS, Tough SC, Chalmers B, Heaman M (2015). Contribution of prepregnancy body mass index and gestational weight gain to adverse neonatal outcomes: population attributable fractions for Canada. BMC Pregnancy Childbirth.

[37] Higgins L, Greenwood SL, Wareing M, Sibley CP, Mills TA (2011). Obesity and the placenta: a consideration of nutrient exchange mechanisms in relation to aberrant fetal growth. Placenta.

[38] Woods L, Perez-Garcia V, Kieckbusch J, Wang X, DeMayo F, Colucci F (2017). Decidualisation and placentation defects are a major cause of age-related reproductive decline. Nat Commun.

[39] Gong Y, Xu Y, Wan K, Wang Y, Zeng L, Zou K (2023). A prospective analysis of optimal total weight gain ranges and trimester-specific weight gain rates for Chinese pregnant women. BMC Pregnancy Childbirth.

[40] Oken E, Taveras EM, Kleinman KP, Rich-Edwards JW, Gillman MW (2007). Gestational weight gain and child adiposity at age 3 years. Am J Obstet Gynecol.

