# Supplementary material for: Maternal age-specific risks for adverse birth weights according to gestational weight gain: a prospective cohort in Chinese women older than 30
Source: BMC Pregnancy Childbirth. 2024 Jan 5;24:36. doi: 10.1186/s12884-023-06231-y (PMC10768087; doi:10.1186/s12884-023-06231-y)
Supplement: Supplementary file 1 — Supplementary Material 1 [file 12884_2023_6231_MOESM1_ESM.docx]

**Figure S1.** The flow chart of the study participants

Abbreviations: GWG, gestational weight gain

**Table S1.** General characteristics of the study participants according to gestational weight gain

| **Characteristics** | **GWG according to IOM guidelines^a^** | | | ***P-value*** |
| --- | --- | --- | --- | --- |
|  | **Below**  **（n=5448）** | **Within**  **(n=8207）** | **Above**  **(n=6199)** |  |
| **Mothers** |  |  |  |  |
| Maternal age (years) | 34.0 (32.0, 36.0) | 33.0 (32.0, 36.0) | 33.0 (32.0, 36.0) | <0.0001 |
| Pre-pregnancy BMI (kg/cm^2^)^b^ | 20.9 (19.5, 22.2) | 21.0 (19.5, 22.7) | 23.0 (20.4, 24.6) | <0.0001 |
| Gestational weight gain (kg) | 9.0 (7.0, 10.5) | 13.0 (11.5, 14.0) | 16.0 (13.5, 18.0) | <0.0001 |
| Gestational diabetes mellitus (n (%))^c^ | 2271 (24.3) | 2013 (26.8) | 909 (30.5) | <0.0001 |
| Preeclampsia (n (%)) | 71 (1.3) | 106 (1.3) | 172 (2.8) | <0.0001 |
| Caesarean delivery (n (%)) | 3501 (64.3) | 5621 (68.5) | 4601 (74.2) | <0.0001 |
| **Newborns** |  |  |  |  |
| Gestational age at delivery (weeks) | 39.0 (38.4, 39.6) | 39.1 (38.7, 39.9) | 39.3 (38.9, 39.9) | <0.0001 |
| Gender, females (n (%)) | 2598 (47.7) | 4062 (49.5) | 2980 (48.1) | 0.08 |
| Birth length (cm) | 49.0 (48.0, 50.0) | 50.0 (49.0, 51.0) | 50.0 (49.0, 51.0) | <0.0001 |
| Birth weight (cm) | 3168 (2920, 3420) | 3300 (3040, 3560) | 3420 (3160, 3695) | <0.0001 |
| Preterm birth (n (%)) | 372 (6.8) | 315 (3.8) | 194 (3.1) | <0.0001 |
| Macrosomia (n (%)) | 86 (1.6) | 306 (3.7) | 534 (8.6) | <0.0001 |
| Large for gestational age (n (%)) | 183 (3.4) | 571 (7.0) | 838 (13.5) | <0.0001 |
| Small for gestational age (n (%)) | 472 (8.7) | 441 (5.4) | 220 (3.6) | <0.0001 |

Data are expressed as median (25%, 75%) or n (%)

^a^ Women were categorized into three groups based on 2009 IOM guidelines [1]

b Pre-pregnancy BMI was based on WHO Asians [2]

^c^ For GDM, GWG was calculated before gestational week 24

Abbreviations: GWG, gestational weight gain; IOM, Institute of Medicine

**Table S2.** Age-specific association between gestation weight gain rate in the second and third trimesters and large-for-gestational-age according to the IOM guidelines

| **GWG rate** | **Total^a^**  **n=12801** | |  | **31-34 yr^b^**  **n=7865** | |  | **≥35 yr^c^**  **n=4936** | |
| --- | --- | --- | --- | --- | --- | --- | --- | --- |
|  | **Crude OR (95% CI)** | **Adjusted OR**  **(95% CI)** |  | **Crude OR (95% CI)** | **Adjusted OR**  **(95% CI)** |  | **Crude OR (95% CI)** | **Adjusted OR**  **(95% CI)** |
| **Overall^d^** |  |  |  |  |  |  |  |  |
| Below | 0.64 (0.49, 0.82) | 0.62 (0.47, 0.81) |  | 0.42 (0.27, 0.63) | 0.42 (0.27, 0.63) |  | 0.88 (0.61, 1.25) | 0.86 (0.60, 1.23) |
| Within | 1.00 | 1.00 |  | 1.00 | 1.00 |  | 1.00 | 1.00 |
| Above | 2.39 (2.05, 2.79) | 1.98 (1.69, 2.33) |  | 2.46 (2.02, 3.02) | 2.03 (1.66, 2.51) |  | 2.28 (1.79, 2.92) | 1.90 (1.48, 2.45) |
| **Underweight^e^** |  |  |  |  |  |  |  |  |
| Below | 0.35 (0.12, 0.90) | 0.35 (0.12, 0.91) |  | 0.38 (0.11, 1.07) | 0.37 (0.10, 1.05) |  | 0.30 (0.02, 2.37) | 0.30 (0.01, 2.48) |
| Within | 1.00 | 1.00 |  | 1.00 | 1.00 |  | 1.00 | 1.00 |
| Above | 1.86 (0.93, 3.72) | 1.88 (0.93, 3.78) |  | 1.90 (0.88, 4.09) | 1.94 (0.89, 4.20) |  | 1.74 (0.32, 9.64) | 1.80 (0.32, 10.04) |
| **Normal weight^e^** |  |  |  |  |  |  |  |  |
| Below | 0.66 (0.48, 0.90) | 0.65 (0.47, 0.89) |  | 0.41 (0.24, 0.67) | 0.42 (0.24, 0.68) |  | 0.95 (0.62, 1.43) | 0.94 (0.61, 1.44) |
| Within | 1.00 | 1.00 |  | 1.00 | 1.00 |  | 1.00 | 1.00 |
| Above | 1.99 (1.65, 2.42) | 2.01 (1.66, 2.45) |  | 2.00 (1.57, 2.57) | 1.99 (1.56, 2.55) |  | 2.01 (1.48, 2.75) | 2.01 (1.48, 2.76) |
| **Overweight/Obese^e^** |  |  |  |  |  |  |  |  |
| Below | 0.79 (0.44, 1.37) | 0.77 (0.43, 1.34) |  | 0.57 (0.20, 1.38) | 0.56 (0.20, 1.36) |  | 0.96 (0.45, 1.95) | 0.92 (0.43, 1.87) |
| Within | 1.00 | 1.00 |  | 1.00 | 1.00 |  | 1.00 | 1.00 |
| Above | 2.10 (1.51, 3.00) | 2.17 (1.55, 3.10) |  | 2.37 (1.49, 3.98) | 2.39 (1.49, 4.04) |  | 1.85 (1.17, 3.06) | 1.95 (1.23, 3.24) |

^a^ Total: n=12801 (overall), n=1272 (underweight), n=8076 (normal weight), n=3453 (overweight/obese)

b 31-34 yr: n=7865 (overall), n=937 (underweight), n=5038 (normal weight), n=1890 (overweight/obese)

^c^ ≥35 yr: n=4936 (overall), n=335 (underweight), n=3038 (normal weight), n=1563 (overweight/obese)

^d^ Adjusted for maternal age, pre-pregnancy BMI, gestational diabetes mellitus, and preeclampsia

^e^ Adjusted for maternal age, gestational diabetes mellitus, and preeclampsia

Abbreviations: GWG, gestational weight gain; IOM, Institute of Medicine

**Table S3.** Age-specific association between gestation weight gain rate in the second and third trimesters and small-for-gestational-age according to IOM guidelines

| **GWG rate** | **Total^a^**  **n=12801** | |  | **31-34 yrb**  **n=7865** | |  | **≥35 yr^c^**  **n=4936** | |
| --- | --- | --- | --- | --- | --- | --- | --- | --- |
|  | **Crude OR (95% CI)** | **Adjusted OR**  **(95% CI)** |  | **Crude OR (95% CI)** | **Adjusted OR**  **(95% CI)** |  | **Crude OR (95% CI)** | **Adjusted OR**  **(95% CI)** |
| **Overall^d^** |  |  |  |  |  |  |  |  |
| Below | 1.31 (1.08, 1.58) | 1.30 (1.07, 1.58) |  | 1.55 (1.22, 1.97) | 1.53 (1.20, 1.95) |  | 1.00 (0.73, 1.37) | 0.98 (0.71, 1.34) |
| Within | 1.00 | 1.00 |  | 1.00 | 1.00 |  | 1.00 | 1.00 |
| Above | 0.56 (0.47, 0.67) | 0.63 (0.52, 0.75) |  | 0.62 (0.49, 0.77) | 0.69 (0.54, 0.86) |  | 0.47 (0.35, 0.63) | 0.54 (0.40, 0.73) |
| **Underweight^e^** |  |  |  |  |  |  |  |  |
| Below | 1.77 (1.19, 2.65) | 1.73 (1.16, 2.61) |  | 1.87 (1.18, 3.01) | 1.91 (1.19, 3.09) |  | 1.52 (0.71, 3.35) | 1.39 (0.63, 3.12) |
| Within | 1.00 | 1.00 |  | 1.00 | 1.00 |  | 1.00 | 1.00 |
| Above | 0.80 (0.46, 1.34) | 0.81 (0.46, 1.37) |  | 0.76 (0.40, 1.43) | 0.82 (0.42, 1.52) |  | 0.84 (0.28, 2.28) | 0.83 (0.27, 2.26) |
| **Normal weight^e^** |  |  |  |  |  |  |  |  |
| Below | 1.09 (0.86, 1.39) | 1.09 (0.85, 1.39) |  | 1.26 (0.92, 1.70) | 1.24 (0.90, 1.70) |  | 0.90 (0.61, 1.30) | 0.88 (0.59, 1.29) |
| Within | 1.00 | 1.00 |  | 1.00 | 1.00 |  | 1.00 | 1.00 |
| Above | 0.59 (0.47, 0.73) | 0.58 (0.46, 0.72) |  | 0.64 (0.48, 0.84) | 0.63 (0.47, 0.83) |  | 0.51 (0.35, 0.73) | 0.51 (0.35, 0.74) |
| **Overweight/Obese^e^** |  |  |  |  |  |  |  |  |
| Below | 1.44 (0.77, 2.65) | 1.55 (0.83, 2.88) |  | 2.50 (1.11, 5.78) | 2.52 (1.10, 5.92) |  | 0.71 (0.24, 1.82) | 0.78 (0.27, 2.05) |
| Within | 1.00 | 1.00 |  | 1.00 | 1.00 |  | 1.00 | 1.00 |
| Above | 0.71 (0.45, 1.15) | 0.62 (0.39, 1.02) |  | 0.89 (0.48, 1.81) | 0.76 (0.40, 1.57) |  | 0.54 (0.29, 1.08) | 0.49 (0.25, 0.99) |

^a^ Total: n=12801 (overall), n=1272 (underweight), n=8076 (normal weight), n=3453 (overweight/obese)

b 31-34 yr: n=7865 (overall), n=937 (underweight), n=5038 (normal weight), n=1890 (overweight/obese)

^c^ ≥35 yr: n=4936 (overall), n=335 (underweight), n=3038 (normal weight), n=1563 (overweight/obese)

^d^ Adjusted for maternal age, pre-pregnancy BMI, gestational diabetes mellitus, and preeclampsia

^e^ Adjusted for maternal age, gestational diabetes mellitus, and preeclampsia

Abbreviations: GWG, gestational weight gain; IOM, Institute of Medicine
